# Practical Guide to Achieve Rigor and Data Integration in Mixed Methods Research

**DOI:** 10.17533/udea.iee.v42n3e02

**Published:** 2024-11-02

**Authors:** Elisiane Lorenzini, Sandra Patricia Osorio-Galeano, Catiele Raquel Schmidt, Wilson Cañon-Montañez

**Affiliations:** 1 Nurse, PhD. Adjunct Professor. Nursing Department. Universidade Federal de Santa Catarina, Florianópolis, SC, Brazil. E-mail: elisiane.lorenzini@ufsc.br. https://orcid.org/0000-0001-8426-2080 Corresponding Author Universidade Federal de Santa Catarina Nursing Department Universidade Federal de Santa Catarina Florianópolis SC Brazil elisiane.lorenzini@ufsc.br; 2 Nurse, PhD. Full Professor, Faculty of Nursing. Universidad de Antioquia, Medellín, Colombia. E-mail: sandra.osorio@udea.edu.co https://orcid.org/0000-0001-9868-2035 Universidad de Antioquia Faculty of Nursing Universidad de Antioquia Medellín Colombia sandra.osorio@udea.edu.co; 3 Nurse, PhD Candidate. Post Graduate Studies Program in Nursing. Universidade Federal de Santa Catarina, Florianópolis, SC, Brazil. E-mail: catieleenf@gmail.com https://orcid.org/0000-0001-7711-4370 Universidade Federal de Santa Catarina Post Graduate Studies Program in Nursing Universidade Federal de Santa Catarina Florianópolis SC Brazil catieleenf@gmail.com; 4 Nurse, PhD. Full Professor, Faculty of Nursing. Universidad de Antioquia, Medellín, Colombia. E-mail: wilson.canon@udea.edu.co https://orcid.org/0000-0003-0729-5342 Universidad de Antioquia Faculty of Nursing Universidad de Antioquia Medellín Colombia wilson.canon@udea.edu.co

**Keywords:** qualitative research, data analysis, nursing research., investigación cualitativa, análisis de datos, investigación en enfermería., pesquisa qualitativa, análise de dados, pesquisa em enfermagem.

## Abstract

Mixed methods research represents a dynamic approach, that combines quantitative and qualitative perspectives in the same study to answer complex questions, beyond the reach of each method used separately. This type of research is increasingly used in health sciences and in social sciences, where it is possible to identify important contributions to knowledge and the practice, derived from the characteristic integration of this approach. Nevertheless, it important to reiterate the importance of recognizing their own perspectives, methods, rigor criteria, and challenges. This work presents general aspects of the epistemological perspective of mixed methods research, describes basic and advanced designs, forms and possible integration moments of each design, as well as the rigor criteria that guide these types of studies. Graphic elements are presented to facilitate recognizing the structure of each design. Furthermore, a visual tool is introduced denominated “anatomy of mixed methods research”, which seeks to guide researchers regarding each of the key elements in the design and development of this type of research.

## Resumen

La investigación de métodos mixtos representa un enfoque dinámico, que combina la perspectiva cuantitativa y cualitativa en un mismo estudio, para dar respuesta a preguntas complejas, más allá del alcance de cada método utilizado de forma separada. Este tipo de investigación es cada vez más usada en las ciencias de la salud y en las ciencias sociales, donde se pueden identificar importantes aportes al conocimiento y la práctica, derivadas de la integración propia de este enfoque. Sin embargo, es importante reiterar la importancia de reconocer sus propias perspectivas, métodos, criterios de rigor y desafíos. Es así, como en este artículo se presentan aspectos generales de la perspectiva epistemológica de la investigación de métodos mixtos, se describen los diseños básicos y avanzados, las formas y posibles momentos de integración de cada diseño, así como los criterios de rigor que orientan este tipo de estudios. Se presentan elementos gráficos que facilitan el reconocimiento de la estructura de cada diseño. Adicionalmente, se presenta una herramienta visual denominada “anatomía de la investigación de métodos mixtos”, la cual busca orientar a los investigadores frente a cada uno de los elementos clave en el diseño y desarrollo de una investigación de este tipo.

## Resumo

A pesquisa de métodos mistos representa uma abordagem dinâmica que combina a perspectiva quantitativa e qualitativa no mesmo estudo, para responder questões complexas, além do escopo de cada método utilizado separadamente. Este tipo de investigação é cada vez mais utilizada nas ciências da saúde e nas ciências sociais, onde podem ser identificadas importantes contribuições para o conhecimento e a prática, derivadas da integração desta abordagem. Portanto, é importante reiterar a importância de reconhecer as suas próprias perspectivas, métodos, critérios de rigor e desafios. Assim, este artigo apresenta aspectos gerais da perspectiva epistemológica da investigação de métodos mistos, descreve os desenhos básicos e avançados, as formas e momentos possíveis de integração de cada desenho, bem como os critérios de rigor que norteiam este tipo de estudos. São apresentados elementos gráficos que facilitam o reconhecimento da estrutura de cada desenho. Além disso, é apresentada uma ferramenta visual chamada “anatomia da pesquisa de métodos mistos”, que busca orientar os pesquisadores sobre cada um dos elementos-chave no design e desenvolvimento de uma pesquisa deste tipo.

## Introduction

Mixed methods research has undergone important development in recent decades. Its use is increasingly more frequent in social sciences and in health sciences due to a growing number of publications guiding its development not only from the methodological point of view, but also epistemological. This type of research represents a dynamic approach of knowledge that permits generating contributions to theory and practice, from recognizing multiple points of view from qualitative and quantitative perspectives.^1^ Currently, using mixed methods research in the nursing discipline is not considered innovative or controversial,^2^ however, important challenges exist to advance in its maximum potential and in enhancing the rigor required by this type of research. Nursing publications derived from mixed methods research have increased, although to a lesser extent compared with other disciplines, like psychology and education. Younas *et al.,*^3^ presented a literature review on publications of mixed methods research in nursing, calculating a prevalence of 1.89% between 2014 and 2018. Nevertheless, the authors identified the need to strengthen aspects related with rigor and the importance of broadening forms of integration.

An aspect of great importance to advance in the development and application of mixed methods research in nursing is its differentiation from multimethod or multiple methods research. The latter refers to diverse combinations of methods that include more than one data collection procedure. It can include two or more exclusively qualitative approaches, two or more quantitative approaches or a combination of qualitative and quantitative approaches.^4^ It is also necessary to highlight that, although the term triangulation has been related with mixed methods research, currently its use is not recommended in this context, given that the term integration is broader and more coherent with this type of research.^4^

Unlike research with multiple methods, mixed methods research is characterized for combining elements from qualitative and quantitative research to achieve more profound understanding of a phenomenon, than what would be possible using each method separately.^4^ It is one of its greatest strengths and, in turn, a guiding aspect when defining and justifying its use. In this sense, it is worth mentioning that in mixed methods research quantitative and qualitative objectives must be established and that, moreover, a clear intentionality must be present in the integration of these types of studies, given that it constitutes the essential component of mixed methods research and it refers to the mixture, fusion, or comparison of qualitative and quantitative findings during data collection, analysis and interpretation.^5^ This is how integrating quantitative and qualitative data is a central and intentional activity, which can be present at the level of conceptualization, operationalization of design, methods, analysis, interpretation and reporting of findings.^5^

It is important to consider that other aspects exist that turn out essential within mixed methods research, such as:^5^ 1) a clear justification to address the problem and the questions through this type of research, 2) the clear definition of a mixed method design, 3) the detailed description of the sampling procedures, collection and analysis of both qualitative and quantitative data, 4) description of the integration process including moments and strategies, 5) evidence of the integration, and 6) description and explanation of the meta-inferences, as well as the knowledge generated by the integration. Within these types of studies, it is also greatly important to describe the researchers’ skills in quantitative, qualitative, and mixed research, as well as to represent the design with a graphic scheme that clearly indicates the point or points of integration.^6^ These aspects are essential in methodological terms and define, largely, the quality and legitimacy of this type of design, adding to the importance of using a theoretical framework within an epistemological perspective, which defines its scope and purpose.

### Epistemological perspective of mixed methods research.

The research process, independent of its approach, is supported on an epistemological posture that guides its vision of reality and the methods of relating with the object of knowledge. In mixed methods research, the epistemological perspective acquires especial relevance, given that its principal characteristic is the qualitative and quantitative data integration within the same study.^7^ Thus, mixed methods research requires a clear epistemological posture by researchers to promote a reflexive and coherent process throughout the entire research process.

Traditionally, the positivist paradigm and interpretative paradigm have proposed a methodological dichotomy aimed at quantitative or qualitative research.^8^ Thereby, integration in mixed methods research has generated debate and tensions. From a paradigmatic point of view, quantitative research implies a vision of an objective and quantifiable reality, a position by researchers outside this reality and neutrality as one of the principal characteristics of the positivist paradigm. On the other hand, qualitative research supposes a non-quantifiable subjective reality, typical of human experience, where researchers interact with that reality and involve their values through reflexibility, as proposed by the interpretative paradigm.^8^ Mixed methods research, in turn, recognizes that a phenomenon may be studied from both approaches and that the results may be complemented from the individual perspective of each paradigm.^7^ This is how the epistemological foundation of mixed methods designs lies, to a large extent, in pragmatism, where, from a reason of complementarity, perspectives join in the production of knowledge to broaden comprehension of a phenomenon.^9^

The complexity of mixed methods research requires profound comprehension of the assumptions and philosophical positions. In this regard, Onwuegbuzie and Corrigan^10^ propose a three-dimensional model to categorize and organize these assumptions: *dimension 1* refers to the point at which assumptions and philosophical positions emerge. In mixed methods research, an *a priori* or *a posteriori* philosophical framework can be used. Nonetheless, an intermediate point exists where philosophical frameworks emerge iteratively, that is, a researcher can begin with a philosophical framework that evolves as research is conceptualized and planned;^10^*dimension 2* contains the assumptions and philosophical positions that underlie the study. At one end are research philosophies that emerge from the tradition of quantitative or qualitative research and at the other end are the research philosophies developed specifically for studies of mixed methods research. Among them, the following have been described: pragmatism, critical realism, dialectics, complementary strengths, the substantive theory, dialectic pluralism, and critical dialectic pluralism, among others.^10^ And, finally, *dimension 3* that establishes the number of philosophies involved in mixed methods research. In one end are studies that do not consider any philosophy explicitly, *i.e.*, they have an a-paradigmatic stance, and in the other end are studies that involve multiple philosophies and which are compatible with pluralist dialectic postures.^10^

Research based on mixed methods may be placed in any part of the three dimensions, which demonstrates that research philosophies can be mixed or integrated in different ways, thereby, it is necessary to make explicit the way in which they are involved and articulated within the study.^10^ This aspect represents a demonstration of the great potential and possibilities of establishing dialogues of perspectives and generating contributions in different areas and disciplines of knowledge. In this sense, it is worth highlighting critical dialectic pluralism as a stance that favors culturally progressive, responsive, and engaged research that promotes social justice, inclusion, diversity, equity, and social responsibility.^10^^,^^11^ This lens for viewing the reality of research promotes, at the same time, rigorous and ethical research, as it guides methodological decisions responsibly to guarantee the well-being of participants and rigor in the different designs of mixed methods research.^10^^,^^11^

This perspective reaffirms the possibilities of mixed methods research to develop knowledge in the nursing discipline. The epistemological postures characteristic of mixed methods are consistent with the disciplinary philosophical perspective of nursing.^2^ It is important to recognize that nursing knowledge emerges from the integration of different forms of knowledge, from ethical, empirical, and sociopolitical dimensions. Research paradigms imply a way of seeing, interpreting, analyzing, and addressing research phenomena. Hence, quality and rigor have a close relationship with the epistemological perspective. Mixed methods research is based on integration; thus, it must assume a logical philosophical position coherent with this perspective, which allows a critical, reflexive, ethical, and rigorous research process.

### Mixed methods designs and data integration.

**Figure 1:**
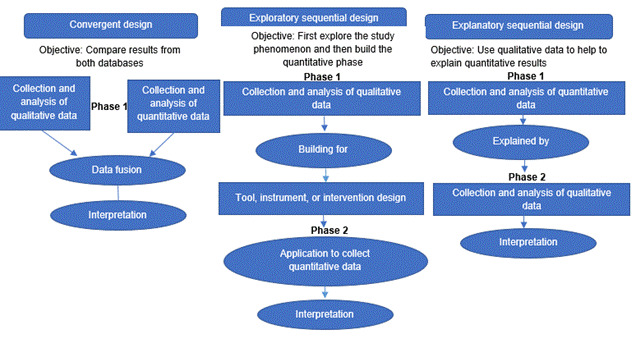
Structure of the principal designs in mixed methods research

Source: Adapted from Creswell JW, Plano-Clark VL. Designing and conducting mixed methods research (3^rd^ ed.). Thousand Oaks, CA: Sage 2017

The three principal or basic mixed methods designs (Figure 1) are already well-defined in the literature. To summarize, here are each of them in function of their main objective: 1) explanatory sequential: that uses qualitative data to explain part of the quantitative results, 2) exploratory sequential: where, first, the study phenomenon is explored and, then, the quantitative phase is constructed, and 3) convergent design: where the objective is to compare the results of both phases, where there can be convergences, divergences, and expansion of the results to respond to the issue of mixed methods research.^5^

Besides the basic designs: convergent, explanatory, exploratory, advanced designs exist: 1) experimental or intervention, 2) case study, 3) participatory: social justice design, and 4) program evaluation/multiphase.^5^ With relation to integration, it is worth noting that it can occur in the design, the method, and in the presentation and interpretation of results. With respect to the type of integration in the method, researchers must indicate the type of mixed methods used, for example: QUANTI + QUALI and specify the type of integration that will be applied to both databases: connection, construction, fusion, or incorporation.^12^

Regarding the type of integration in the results presentation and interpretation, it is possible analyze and present qualitative and quantitative results integrated by: *(a) Narrative*: qualitative and quantitative results together or in separate sessions and an article from the complete study of mixed design; this design was used in the study by Neves *et al.*;^13^ *(b) Data transformation*: convert qualitative data into quantitative or vice-versa and integrate them with data not converted in correlation and comparison terms. For example, in the study by Lorenzini *et al.,*^14^ a database was integrated with another through the sample of participants in a quantitative and qualitative phase, through a validated questionnaire and the completion of open questions, intentionally designed to answer complex questions, within the framework of a convergent design. Additionally, after the qualitative analysis and generation of categories, data were transformed to establish quantitative variables, which were then object of descriptive and inferential analysis; and (c) *Joint display* of qualitative and quantitative data or joint display that includes a theory.^5^^,^^12^ In this design, presenting “*insights”*, inferences, and meta inferences at the end of the results or in the interpretation broadens the discussion. An example of this design is presented in the study by Lorenzini *et al*.^15^

Application of integration principles and practices can promote the use of the strengths of mixed methods. It is quite important to understand that different forms of integration qualify the mixed study and can occur at different moments of the study. Thereby, to demonstrate the integration, it is paramount to discuss the principal quantitative and qualitative findings while the specific study results are related with similar or divergent results in the literature. Further, it is necessary to analyze the issue of mixed methods research and generate meta inferences to, finally, propose explanations for the study phenomenon and formulate recommendations based on the accumulated evidence generated by the study in question, combined with other evidence from the literature.

**Figure 2:**
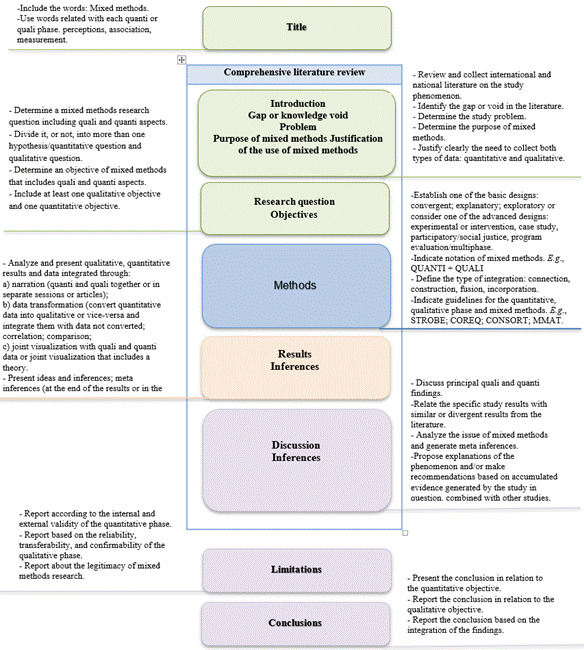
Anatomy of mixed methods research

Source: Elisiane Lorenzini, Universidade Federal de Santa Catarina, Brazil.

Figure 2 presents the tool called “Anatomy of mixed methods research” developed by Professor Elisiane Lorenzini from Universidade Federal de Santa Catarina in Brazil. Using this tool can facilitate identifying each of the stages of mixed methods research and recognizing visually the minimum elements expected in the project description and in the research report.

**Figure 3:**
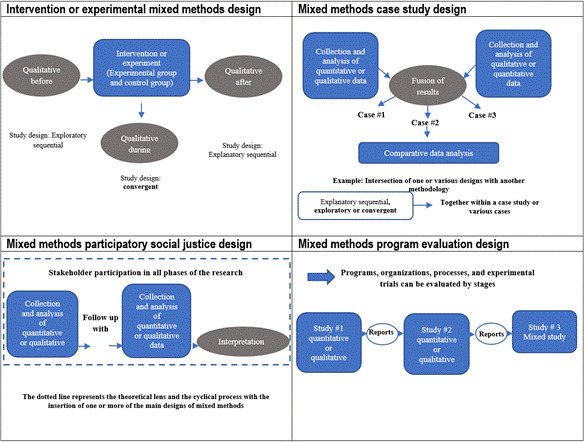
Structure of advanced mixed methods designs

Source: Adapted from Creswell JW, Plano-Clark VL. Designing and conducting mixed methods research (3^rd^ ed.). Thousand Oaks, CA: Sage 2017.

Although convergent, explanatory sequential, and exploratory sequential designs are called basic, they are complex in themselves; the continuous evolution of mixed methods research has allowed developing and presenting new designs. According to Creswell and Plano-Clark,^16^ four types exist denominated Advanced Mixed Methods Designs, which are shown in diagrams in Figure 3 with a brief description, besides examples of the application of each.

### Experimental or intervention mixed methods design

In this design, a quantitative experimental or intervention study was conducted and the collection and analysis of qualitative data was carried out within the framework of a qualitative study. The collection stage of qualitative data can take place in one or more moments during the planning and execution of the intervention; that is, it can occur before, during, or after the intervention, or at all moments. An example of this type of research can be consulted in the study by Jafarpour.^17^

Given the rigor inherent to both research approaches, some procedures must be considered:^5^ 1) determine how the qualitative data will be used in the experiment; 2) determine why the qualitative information is necessary and how it will be used; 3) identify when qualitative data will be collected (before, during, or after the intervention) or during the three phases; 4) conduct the experiment/intervention of the quantitative stage; 5) specify the conceptual model that guides the design; 6) assign participants to the treatment group and to the control group; implement the treatment/intervention proposed; 7) measure variables and results; 8) analyze quantitative data using descriptive and inferential statistics to respond to the quantitative hypothesis; 9) collect and analyze qualitative data if planning to collect them during the experiment to answer questions of qualitative research; 10) perform procedures to integrate quantitative and qualitative data based on the reasons to include qualitative data in an intervention or experimental study; 11) determine how the results improved the experiment, being careful not to interfere in the effectiveness of the intervention carried out; 12) present specific inferences from the use of qualitative results; 13) interpret and reflect about how the qualitative results helped to improve the experiment or, for example, in a randomized clinical trial (RCT). Therefore, the emphasis is on the need to maintain the rigor of each of the designs that are part of a mixed methods research. For the RCTs, the declaration of Consolidated Standards of Reporting Trials (CONSORT) and its extensions propose the critical points that these types of studies must include and which are essential to determine the efficacy or effectiveness of interventions within the framework of a mixed design. This is how these types of studies in their experimental component must be guided by the criteria of this declaration.^18^

These types of advanced designs are related with the basic designs. When researchers decide that qualitative data will be collected prior to the intervention, the basic or principal design used will be exploratory sequential.^5^ Some examples about the reasons for collecting qualitative data before the quantitative or intervention stage can be the need to develop a questionnaire for its subsequent application or to identify pre- and post-trial measures of interest, identify possible participants for rare case studies, understand the context and setting where the trial will be implemented, or to document the need for the intervention. When researchers decide that qualitative data will be collected during the intervention, the basic or principal design used will be convergent.^5^ Some examples that can explain the need to collect during the implementation of the intervention include the importance of understanding the experience of participants about the intervention proposed, identifying possible mediating or moderating factors, understanding participants' experience of facilitators and barriers during the implementation of the intervention, identifying resources that can affect the implementation of the intervention, accessing or verifying the fidelity of the intervention’s procedures.

A third possibility exists and it is that researchers decide to collect qualitative data after the intervention. In this case, the basic or principal design used will be explanatory sequential.^5^ Some examples of why data collection may be necessary after implementing the intervention, include the need to understand why the results occurred, receive feedback from participants, revise or change the intervention, help to explain some variations in the results, examine the sustainability of the effects of the intervention over time, help to explain the mechanisms that may have operated during the trial, or test how the context may have influenced on the results. It is worth noting that in research using mixed intervention methods, researchers must determine at which point will the integration occur or if it will occur in all stages, based on the question of qualitative research to explore. Thereby, the quality of the qualitative study will add value to the study as a whole. 

### Mixed Methods Case Study Design

Mixed designs imply using a basic design (convergent, explanatory sequential, exploratory sequential) within the framework of a case study that can be single or multiple. This design collects both quantitative and qualitative data and the final product is the generation of one or various detailed cases and contextualized beyond a case that contains only quantitative or qualitative data.^5^ Frequently, both types of data are collected simultaneously in a convergent design and, after that, the results are fused, where integration takes place. Comparative analysis of cases is added to this, which constitutes a space for data integration. Some advantages commonly associated with case studies are the possibility of developing profound understanding and practical, individualized and transferable conclusions. In addition, they allow comprehending the complexity of a case, comparing cases from the qualitative and quantitative dimension to represent their variations and knowledge. 

This type of design requires prior experience in procedures related with case studies and with qualitative studies, which lead to selecting the adequate design and defining a single or multiple case. An example of a mixed methods case study conducted by Poth *et al.,*^19^ can be reviewed to broaden understanding of this design. This study sought to assess the impact of competency-based programs in training evaluators in medical education. This design was appropriate due to the need to capture the complexity of the many facets that contribute to an effective learning environment for evaluators. The case was limited by the duration of the course and its participants to generate comprehensive understanding of the experience by the students. The results provided empirical evidence about the impact of these types of training programs and the comparative analysis of effective performance to train evaluators at graduate level. The study indicated the need to understand who the students are and how to facilitate significant interactions among those involved in the course, among other aspects. 

### Mixed Methods Participatory - Social Justice Design

This involves developing a central design within a theoretical framework or conceptual participatory and/or social justice framework, such as - for example - a feminist or participatory theory.^5^ First, quantitative and qualitative data are collected and analyzed in separate studies. Thereafter, the results are interpreted, wherein integration is produced. In this type of study, ideally, the principal researcher must have extensive knowledge of the theory that illuminates the study as a whole. The main objective is to empower participants to generate needed changes with respect to their own problems identified with the aid from researchers during the study. One of the strengths that can be highlighted from this design is that it permits articulating any basic or principal design with a mixed methods participatory social justice design.^5^ The study by Greysen^20^ is an example in which it can be identified that it started with participation from interested parties by collecting data via interviews and focal groups. Another example is the study by NeMoyer^21^ who began the research as of the data from a survey, during a quantitative stage, followed by other stages that included collecting qualitative and quantitative data in a multiphase design.

Another strength of these types of designs is that they can be more easily accepted among groups of interested parties, as researchers help to generate changes in practice and to promote empowerment based on results useful to participants, communities, and those responsible for public policies.^5^ One of the principal challenges in this design is the need for researchers to be experienced in identifying the theoretical frameworks and previously familiarized with them, and who have the ability to communicate the framework to the participants to implement it throughout the study (formulation of the problem, results collection, analysis, and interpretation). In this same sense, researchers face the challenge of developing trust with the participants, foster in them the desire to change, which is necessary for the participatory methodology, besides having sufficient skills and sensitivity to conduct research while respecting the culture.^5^

### Mixed Methods Program Evaluation Design

It is a design that includes one or more central designs added to the evaluation of an intervention or a program during a period of time, with hopes of guiding its development, enhancement, or adaptation.^5^ These studies can be multiphase, for example, they can include collecting and analyzing qualitative and quantitative data in separate studies. Thus, these prior studies could support a third study, for example, involving one or more of the principal designs in the phases of an evaluation procedure. These research normally center on assessing the success of a program, process, activity, or intervention.^5^ The strengths of this design include the flexibility to employ any element of mixed methods to answer interconnected research questions, ability to publish results from individual studies while still contributing to the broader study, and ability to build a framework to conduct multiple iterative studies over several years. In addition, it can provide multiple results for diverse objectives, such as, for example, evidence about the practices. ^(^^5^

With respect to the challenges of this design, one of the main ones is to meaningfully connect individual studies, combined with the principal designs of mixed methods and other studies, as well as to establish the dynamics of the research team and maintain it.^5^ Other challenges imply involving the participants, managing resources, making more than one presentation to the Research Ethics Committee, as well as translating the knowledge to apply it in the practice.^22^

As seen, principal designs alone or in their intersection with complex frameworks, which account for the methodological rigor inherent to each method, can represent mixed methods studies of high scientific value. Regarding the design, it must be mentioned that an interesting perspective has been described, which classifies designs as typological, where basic and complex designs described are found, as well as the interactive or dynamic designs.^5^^,^^23^ In these last ones, the design is understood as a continuous, interactive, and complex process that demands researchers continuous comparison of the components during the research development, to verify that they adapt to each other or, if necessary, re-establish or adjust them.^23^ In all cases, describing a type of design within mixed methods research is a central aspect, hence, it must be clearly detailed and articulated coherently with the mixed nature of the research problem, questions, and study objectives. 

### Rigor in mixed methods research

A starting point that permits addressing the perspective of rigor within the mixed method is to emphasize the need to maintain rigor in each of its components as an aspect that underpins the integration and complementarity of the method.^24^ Nevertheless, if the components are not evaluated as a whole, the study will not provide an analysis of the rigor from the integration characteristic of the mixed perspective. Currently, widely disseminated tools exist that guide the assessment of the quality of mixed methods research; the best known of these is the Mixed Methods Appraisal Tool, which evaluates five categories:^25^ 1) justification of adopting mixed methods; 2) integration between quantitative and qualitative components; 3) interpretation of the integration between quantitative and qualitative data; 4) presentation of divergences between quantitative and qualitative results; and 5) compliance of the methodological rigor of each research approach. This tool has been adapted and validated in different contexts and languages and serves as a guide that largely oversees the quality of research under this approach.^26^

It is worth mentioning that it is useful to consider checklists or specific tools to assess methodological quality to verify the rigor criteria of the qualitative and quantitative components, according to the methodological design of each. However, these tools by themselves do not reflect the rigor of mixed method research where the study needs to be valued as a whole. In this sense, it is important to highlight that research rigor implies profound comprehension of the concepts that define it and the strategies that contribute to its enhancement, recognizing the challenge this type of research entails by combining philosophical paradigms and a great diversity of methodologies. Given the perspective of complementarity, the rigor of one component directly affects the quality of the other and can, ultimately, affect the results and legitimacy of the meta-inferences - understood as the inferences made from the qualitative and quantitative findings integrated in mixed methods research.^24^

Lincoln and Guba^27^ developed concepts that have helped to define the criteria to judge qualitative and quantitative research from a perspective coherent to each approach. In this same line, Koch^28^ proposed an equivalence in the dimensions from which rigor criteria can be established in qualitative and quantitative research, from the principles that guarantee the quality of the studies. This is how methodological rigor can be expressed in both approaches from: the *value of truth*, *applicability*, *consistency*, and *neutrality.*

In turn, in mixed methods research, the dimensions of quality as a whole are complex, but widely accepted perspectives exist with clear connections with the general criteria of research quality. Two dimensions exist of great importance within the rigor of mixed methods research, according to Eckhardt and DeVon^24^, these are: (i) *quality of the inference,* determined by the quality of the design and by the interpretative rigor; this last aspect refers to the degree to which the interpretations of the data are derived directly from the results obtained and is characterized by the interpretative consistency, theoretical consistency, interpretative agreement, interpretative distinction, and integrative effectiveness;^24^ and, (ii) *Legitimation* that refers to the researcher’s capacity to make credible, reliable, and confirmable inferences.^24^^,^^29^ Legitimation has to do with aspects, like the sample and the sampling, the degree to which the subjects' point of view is presented, minimization of the weaknesses of each method, the paradigmatic mix, commensurability, defined as a third point of view that extends beyond the qualitative or quantitative points of view by themselves, among others.^22^^,^^27^ Legitimation is central aspect of the quality of mixed methods research, it is not centered on evaluating the qualitative or quantitative methods, but on a joint vision of quality. In terms of quality and the rigor of mixed methods research, highly important perspectives have also been described, like the *validation framework* and the *quality framework*.^30^^,^^31^

The *validation framework* includes traditional rigor elements, like validity, reliability, and credibility, as well as other aspects, such as the researchers’ prior knowledge about the concept of interest and the inferential consistency, which refers to whether the conclusions drawn are appropriate given the study’s methodological options. The validation framework should be used as research is planned and carried out to ensure the quality of the data and inferences.^30^ Within this framework, collaborative work in research teams highlights a special importance as an element that favors the quality of mixed methods. This type of research is strengthened by a team of researchers with diverse backgrounds and strengths in quantitative, qualitative, and mixed research in which each member contributes from their experience to promote the rigor, respecting the contribution by each method.^22^

Lastly, the *quality framework*^31^, proposes as its center, the quality of the inference based on domains, such as the quality of planning, design quality, data quality, interpretative rigor, transferability of the inference, quality of the reports, capacity for synthesis, and utility. These frameworks or dimensions of the quality of mixed methods research are coherent with each other and reaffirm the need to understand the research quality and rigor from each method and as a whole. Applying tools and checklists facilitates assessing quality, but its foundation lies in understanding the dimensions of rigor, its expression, and strategies to favor it. Table 1 presents the rigor criteria according to each research approach, as well as some of the strategies that promote them. It is identified that the rigor perspectives of mixed methods research connect with traditional rigor dimensions and that aspects related with integration become a central element of the quality of mixed methods research.

**Table 1:** Strategies to favor rigor according to type of research

Rigor criteria	Qualitative research	Quantitative research		Mixed methods research
Value of truth	Credibility Use of synthesis and repetition, textual transcriptions, triangulation techniques during interviews by researchers, validation of results findings with participants.	Internal validity Control of confounding and selection biases. Clear definition of inclusion and exclusion criteria.	1	Quality of the inference Justification of the use of the design and coherence with the research question and objective. Clarity and fidelity of the type of mixed design used and explicit definition of the type and moment of integration. Clarity with respect to the variables and decisions regarding their integration in the qualitative component.
Applicability	Transferability Theoretical sampling, Detailed archive of audios, transcripts, memos, matrices, and diagrams. Constant comparison of data and contrast with the literature. Description of the context and the characteristics of the participants.	External validity Sampling strategies and calculation of sample size.		Legitimation Definition of the type of samples (independent or inter-dependent) and the type of sampling in each component. Sampling calculation. Detailed methods to obtain and analyze data, visibility of participants’ voices. Description and evidence of the integration process.
Consistency	Reliance Triangulation. Researcher’s reflexibility in the process.	Reliability Use of questionnaires and instruments guided by the objectives. Use of validated and reliable instruments to apply in the context.		Validation framework Validity, reliability, applicability. Researchers’ prior experience and track record, inferences consistent and coherent with methods and data.
Neutrality	Confirmability Textual transcription of the interviews, application of confirmation and synthesis techniques during the interviews. Triangulation by researchers.	Objectivity Application of questionnaires through self-completion and in opportune spaces and moments. Masking in experimental studies.		Quality framework Justification of the use of the design and coherence with the research question and objective. Clarity and fidelity of the type of mixed design. Explicit definition of the form and location of the integration.

Finally, it may be concluded that mixed methods research enables the dialogue of perspectives, from the complementarity of the various methodologies, both quantitative and qualitative, to respond to complex questions, beyond the reach of using each type of research separately. This allows contributing to the generation of dynamic scientific knowledge sensitive to the social and cultural contexts in which the research process takes place. In this regard, it is fitting to recognize that this type of research implies great complexity derived from the integration and generation of meta inferences. Thus, among the main challenges, the clarity and pertinence of its theoretical and epistemological support and the rigor in each of the quantitative, qualitative and mixed methods as a whole stand out. The aspects presented seek to provide practical elements that facilitate the process and guide the development of these types of studies. 
